# Regulatory T Cell Activity and Signs of T Cell Unresponsiveness in Bovine Paratuberculosis

**DOI:** 10.3389/fvets.2014.00020

**Published:** 2014-10-21

**Authors:** Jonathan A. Roussey, Juan P. Steibel, Paul M. Coussens

**Affiliations:** ^1^Comparative Medicine and Integrative Biology Program, Michigan State University, East Lansing, MI, USA; ^2^Department of Animal Science, Michigan State University, East Lansing, MI, USA

**Keywords:** Johne’s, paratuberculosis, regulatory T cell, Treg, anergy

## Abstract

Johne’s disease, caused by infection with *Mycobacterium avium* subspecies paratuberculosis (MAP), is a wasting disease of ruminants displaying a long subclinical stage of infection followed by clinical disease characterized by severe diarrhea, wasting, and premature death. Immunologically, subclinical disease is characterized by a Th1 response effective at controlling intracellular infections such as that caused by MAP. In late subclinical disease, the Th1 response subsides and a non-protective Th2 response becomes prominent. One hypothesis for this shift in immune paradigm is that a population of MAP-reactive regulatory T cells (Tregs) develops during subclinical infection, limiting Th1-type responses to MAP antigens. To investigate this, we sought to accomplish the following: (1) determine if CD4^+^CD25^−^ T cells exposed to MAP-infected macrophages develop a Treg phenotype, (2) develop a method to expand the relative abundance of Tregs in bovine peripheral blood lymphocyte populations, and (3) identify functional activities of expanded Tregs when combined with autologous peripheral blood mononuclear cells (PBMCs) and live MAP. We found that CD4^+^CD25^−^ T cells exposed to MAP-infected macrophages from cows with Johne’s disease do not show signs of a Treg phenotype and appear unresponsive to MAP antigens. A method for Treg expansion was successfully developed; however, based on results obtained in the subsequent functional studies it appears that these Tregs are not MAP-specific. Overall, it seems that T cell unresponsiveness, rather than Treg activity, is driving the Th1-to-Th2 immune shift observed during Johne’s disease. Further, we have successfully developed a method to enrich non-specific bovine Tregs that exert suppressive effects against Th1 cytokine production.

## Introduction

Johne’s disease, a chronic wasting disease of ruminants, is caused by infection with *Mycobacterium avium* subspecies *paratuberculosis* (MAP). Johne’s disease (paratuberculosis) is characterized by persistent diarrhea, progressive wasting, and premature death (1). Between 40 and 68% of all U.S. dairy herds contain some incidence of Johne’s disease (2, 3), leading to a substantial economic burden that has been estimated to be as high as $1.5 billion annually for the U.S. dairy industry alone (4). Exacerbating the widespread incidence of Johne’s disease is the long subclinical stage of infection, which frequently lasts from 2 to 5 years (2). During this time, infected animals show little or no signs of disease and often go undetected for years while continually spreading MAP in their feces.

*Mycobacterium avium* subspecies *paratuberculosis* is transmitted primarily via the fecal–oral route and upon ingestion colonizes tissue-resident macrophages within the ileum of the small intestine (5, 6). MAP-infected cattle initially develop a Th1-like (pro-inflammatory and cytotoxic) immune response that can be effective at limiting spread of the infection. During the course of subclinical disease, however, the Th1 response wanes and a Th2-like (humoral) response becomes predominant in some animals (5, 7–9). A Th2 response is not effective at controlling intracellular infections, however, and the spread of MAP within the host during this time may ultimately lead to progression of the infection and clinical disease, usually leading to premature culling of the infected cow.

It is not currently known why the Th1-to-Th2 immune balance shifts away from an effective Th1 response and toward an unproductive Th2 response (7). One possibility is that a population of regulatory T cells (Tregs) develops in response to chronic low level stimulation with MAP antigens and subsequently functions to limit effector T cell responses to MAP (10). Thymic-derived Tregs are largely thought to control autoimmune reactivity, but another population of induced Tregs has been shown to develop and respond to many infectious agents, including *Mycobacterium tuberculosis* (11–14). Both types of Tregs are characterized by expression of the T helper cell co-receptor CD4, the interleukin (IL) 2 receptor alpha chain, CD25, and the transcription factor Forkhead box protein 3 (FOXP3). Induced Tregs are known to exert their immunomodulatory effects through production of IL10 and/or transforming growth factor beta (TGFβ). Neutralization of IL10 in peripheral blood mononuclear cells (PBMCs) from MAP-infected cows results in an increase in recall responses and production of interferon gamma (IFNγ) (15). Further, removal of CD4^+^ or CD25^+^ cells from PBMCs of MAP-infected cattle prior to MAP antigen stimulation also enhances production of IFNγ mRNA in response to stimulation with MAP antigens (10, 15). There is a significant increase in the relative expression of FOXP3 mRNA in ileal tissues from cows with subclinical Johne’s disease as compared to healthy controls (10), and recent evidence suggests that PBMCs from MAP-infected cows contain more CD4^+^CD25^+^FOXP3^+^ cells than similar populations from uninfected controls (16). One possibility for induction of Tregs by MAP is the lack of proper co-stimulation between naïve T cells and macrophages. MAP infection of host macrophages results in decreased CD40:CD40 ligand interaction between MAP-infected macrophages and T cells (17). Failure to properly co-stimulate T cells has been demonstrated to lead to induction of a Treg phenotype (18–20). Thus, existing evidence suggests that Tregs play a role in MAP infection and could be one factor leading to the shift in immune response and progression of Johne’s disease.

Presently, direct evidence for development of functional Tregs responding to MAP antigens or MAP-stimulated effector or cytotoxic T cells is lacking. Thus, we sought to examine the functional nature of Tregs from MAP-infected cows and to determine if improper co-stimulation by MAP-infected macrophages could lead to induction of a Treg phenotype in naïve T cells. Specifically, we sought to accomplish three main goals. First, we brought autologous CD4^+^CD25^−^ T cells – a population that contains, crucially, naïve T cells (in addition to memory and unactivated effector T cells) into contact with MAP-infected monocyte-derived macrophages (MDMs) to determine if the CD4^+^CD25^−^ T cells would develop a Treg phenotype under these conditions. Second, we developed an assay to expand the relative abundance of Tregs within a peripheral blood lymphocyte population for use in functional studies. Finally, we studied the effects of these expanded Tregs on PBMC responses to stimulation with live MAP. Altogether, these experiments were designed to help increase our understanding of both the development and function of Tregs in the context of MAP infection and Johne’s disease.

## Materials and Methods

### Animal cell donors and Johne’s disease testing

Nineteen adult Holstein cows (age > 2 years) were used in the current study. The cows were housed and maintained in a typical commercial two-milking-per-day dairy operation. There were eight healthy controls, eight cows with clinical Johne’s disease, and three cows with subclinical Johne’s disease. Johne’s disease status was verified by serum ELISA (positive result indicated by OD ≥ 1.0) and fecal PCR using insertion sequence 900 (IS900). Diagnostic tests were performed by commercial testing firm Antel Biosystems, Inc. (Northstar Cooperative, Lansing, MI 48910). ELISA readings were taken at least twice for each animal over a 3-month period to confirm disease status. Subclinical infected animals were diagnosed using a modified Bovigam assay in which *Mycobacterium bovis* antigens are replaced with MAP antigens (purified protein derivative of johnin: PPDj; National Animal Disease Center, Ames, Iowa) and IFNγ release in response to these antigens is measured by ELISA. All protocols were reviewed and approved by the Michigan State University Animal Use and Care Committee.

### Isolation of peripheral blood mononuclear cells

Peripheral blood mononuclear cells were isolated from blood samples aseptically drawn into acid–citrate–dextrose (ACD) Vacutainer Blood Collection Tubes (Becton Dickinson catalog #364606) by Percoll gradient density centrifugation (density = 1.084 g/mL). Blood samples were centrifuged for 20 min at 2200 rpm at room temperature. Buffy coats were removed and added to 50-mL conical tubes containing 10 mL Percoll overlayed with 20 mL phosphate buffered saline (PBS), followed by centrifugation at 1380 rpm for 41 min at room temperature. PBMCs were removed from the Percoll/PBS interface and washed twice with 50 mL PBS followed by centrifugation (5 min, 1800 rpm, room temperature). Cells were finally suspended in PBS at approximately 2 × 10^7^ PBMCs/mL for counting and use in subsequent experimentation.

### Generation of monocyte-derived macrophages and MAP infection

Approximately 3.33 × 10^6^ PBMCs were plated per well in 24-well plates with Roswell Park Memorial Institute (RPMI) complete media (RPMI plus 10% fetal bovine serum, 1% penicillin/streptomycin, and 1% fungizone, pH 7.4) and allowed to settle and adhere for 4 h. Non-adherent cells were washed away with three washes of warm PBS, and adherent monocytes (5 × 10^5^ per well on average) were allowed to differentiate into MDMs for 3 days at 38°C with 5% CO_2_. MDMs were then infected with live MAP (American Type Culture Collection Strain #19698) at a multiplicity of infection (MOI) of 20 bacteria per macrophage. This strain of MAP has previously been shown to infect 40–80% of bovine MDMs (21, 22). Further, this strain of MAP is known to be unaffected by penicillin and streptomycin (Sreevatsan S, personal communication), and as such antibiotics were not removed from cultures prior to infection. As an added benefit, this further minimized the risk of unwanted contamination. Infection was allowed to progress for 4 h at which time supernatant was removed and MDMs were washed three times with warm PBS. Processing of phagocytosed MAP was allowed to proceed for an additional 20 h.

### Antibodies and cell sorting

The 5 × 10^7^ PBMCs were labeled with mouse monoclonal antibodies raised against bovine CD4 [isotype IgG1, Washington State University Monoclonal Antibody Center (WSUMAC) catalog #BOV2012, clone CACT138] and bovine CD25 (isotype IgG3, WSUMAC catalog #BOV2076, clone LCTB2A) at a dilution of 10 μg antibody per 5 × 10^7^ PBMCs in 500 μL staining buffer (PBS with 2% horse serum, 10% ACD, and 0.09% sodium azide) for 30 min at 4°C in the dark. Washing buffer (4 mL, PBS with 10% ACD and 0.09% sodium azide) was added to each sample and samples were centrifuged for 5 min at 1500 rpm and 4°C. Cells were then incubated with secondary antibodies raised against mouse IgG1 (goat anti-mouse, Tri-Color, Life Technologies SKU# M32006) and mouse IgG3 (goat anti-mouse, Alexa Fluor^®^ 488, Life Technologies SKU# A-21151) for 30 min in 500 μL staining buffer at 4°C in the dark. Washing buffer (4 mL) was added to each sample and samples were centrifuged for 5 min at 1500 rpm and 4°C. Cells were diluted to a final concentration of 5 × 10^6^ PBMCs/mL in Hank’s balanced salt solution (HBSS) and kept at 4°C in the dark until cells were sorted. Cell sorting was performed with a Becton Dickinson Influx Cell Sorter. Cells were first gated on lymphocytes, and within the lymphocyte population both CD4^+^CD25^−^ lymphocytes and CD4^+^CD25^+^ lymphocytes were collected. For each cow, 5 × 10^5^ CD4^+^CD25^−^ lymphocytes and 7.5 × 10^5^ CD4^+^CD25^+^ lymphocytes were collected for use in cell culture. Cell populations were at least 98% pure following cell sorting.

### CD4^+^CD25^−^ T cell stimulation

CD4^+^CD25^−^ lymphocytes (2.4 × 10^5^) were co-cultured with 4-day-old nil- or MAP-infected MDMs in RPMI complete media for 18 h in 24-well plates at 38°C and 5% CO_2_. Non-adherent cells were collected from the culture and plates were washed twice with warm PBS to remove remaining non-adherent cells. Non-adherent cells were then centrifuged for 5 min at 1800 rpm and 4°C, and RNA was isolated using a Qiagen RNeasy Plus Mini Kit (catalog #74134) according to manufacturer’s instructions. RNA quality was assessed in all cases using an Agilent 2100 Bioanalyzer. A minimum RNA Integrity Number (RIN) of 7.5 was consistently observed and considered acceptable for downstream analyses.

### CD4^+^CD25^+^ T cell enrichment

The 3 × 10^5^ CD4^+^CD25^+^ lymphocytes were cultured with 4-day-old MAP-infected MDMs in RPMI complete media plus enrichment cocktail [1 nM rapamycin (Sigma-Aldrich catalog #R03950-1MG), 5 ng/mL recombinant bovine IL2 (Kingfisher Biotech, Inc. catalog #RP0026B-005), and 2.5 ng/mL purified human transforming growth factor beta 1 (BD Biosciences catalog #354039)] for 8 days at 38°C and 5% CO_2_, with enriched media being refreshed every 72 h.

### Co-culture of PBMCs with enriched CD4^+^CD25^+^ lymphocytes and live MAP

Autologous PBMCs were freshly isolated and 7.5 × 10^5^ PBMCs were combined with or without live MAP (MOI = 2.5) and with or without 3.75 × 10^5^ enriched CD4^+^CD25^+^ T cells. Cultures incubated for 18 h at 38°C and 5% CO_2_ at which point they were collected and RNA was isolated as described above.

### Flow cytometry and data analysis

Peripheral blood mononuclear cells were labeled with anti-CD4 (WSUMAB catalog #BOV2012) and anti-CD25 (WSUMAB catalog #BOV2076) primary antibodies and subsequently labeled with Tri-Color (Life Technologies SKU #M32006) and Alexa Fluor^®^ 488 (Life Technologies SKU #A-21151) as described previously. The eBiosciences FOXP3 fixation/permeabilization buffer was used to permeabilize cells according to manufacturer’s instructions, followed by 45 min incubation at 4°C in the dark with eBiosciences anti-mouse FOXP3-RPE antibody (clone FJK-16s). Rat IgG2a-RPE isotype was used as a control. Using a BD FACSCalibur Flow Cytometer, 50,000 events were collected and analyzed by using a subsequent gating strategy targeting lymphocytes, followed by CD4^+^ lymphocytes, then CD25^hi^ CD4^+^ lymphocytes, and finally FOXP3^+^CD25^hi^CD4^+^ lymphocytes. These cells were then compared to the total number of lymphocytes present to determine the relative abundance of Tregs within a population.

### cDNA conversion and quantitative real-time polymerase chain reaction

RNA (200 ng) was reverse-transcribed into copy DNA (cDNA) using the Applied Biosystems high-capacity cDNA reverse transcription kit (Life Technologies catalog #4387406) according to the manufacturer’s instructions. Quantitative real-time polymerase chain reaction (qPCR) was performed using Applied Biosystems 7000 and 7500 Real-Time PCR systems in duplicate for each sample using Power SYBR Green master mix (Life Technologies catalog #4367659), with 20 μL reaction volumes. Transcripts examined and primer sets used are summarized in Table 1.

**Table 1:** **qPCR primers**.

Gene	Forward Primer (5′ → 3′)	Reverse Primer (5′ → 3′)
CTLA4	CTGTGCTGGGACCTACATGG	TTCCTCTGGAGGTGCCAATG
FOXP3	CACAACCTGAGCCTGACAA	TCTTGCGGAACTCAAACTCATC
GAPDH	AGGAGCACGAGAGGAAGAGT	TTCTCAGTGTGGCGGAGATG
GATA3	AGGTACGTCCTGTGCAAACT	AGACAGGGTCTCCATTGGCA
GITR	CCTGTTTCCCGGAAACAAGAC	CCAGAGAGAGGATGACGATGGT
IFNγ	TCTGGTTCTTATGGCCAGGG	GAAGAGAGGCCCACCCTTAG
IL1A	TTGGTGCACATGGCAAGTG	GCACAGTCAAGGCTATTTTTCCA
IL10	GCTGTATCCACTTGCCAACC	ATCCAGCAGAGACTGGGTCA
IL12P40	CACCCCGCATTCCTACTTCT	TGGCATGTGACTTTGGCTGA
IL17A	CATCTCACAGCGAGCACAAG	CCACCAGACTCAGAAGCAGT
IL2	CAAGCTCTACGGGGAACACA	TAGCGTTAACCTTGGGCACG
IL23	AGCTCTCACAGCAACTCTGC	TGTCCCATTGGTAGGTGTGC
IL4	GGCGGACTTGACAGGAATCT	TTGTGCTCGTCTTGGCTTCA
IL6	GGCTCCCATGATTGTGGTAGTT	GCCCAGTGGACAGGTTTCTG
PFN1	CATGGACCACGGTCTCTTGAA	GGTGAGGCAAGCATTTGACC
RORC	GAGTTCGCTAAGAGGCTCCC	TCCATGGCTCCTGCTTTGAG
TBX21	ACCACCTGTTGTGGTCCAAG	ATCCGGTAATGGCTGTTGGG
TGFβ1	CGAGCCCTGGACACCAACTAC	CCGGAAGTCAATGTAGAGCTGA

### qPCR data interpretation and statistical analysis

Each gene was calibrated against an internal control [*GAPDH*; primer validation performed prior to qPCR for genes of interest. Primer validation was performed by obtaining Ct values for different proposed control genes across all experimental conditions tested. No significant differences (*n* = 3) between experimental conditions were seen in Ct values for GAPDH; as such this was chosen as an internal control], followed by comparing samples from test-positive cows against pooled averages from test-negative cows for each particular culture condition. A linear mixed model analysis was used for statistical analysis as described elsewhere (23); *p* < 0.05 was considered significant.

## Results

### CD4^+^CD25^−^ T cells from cows with subclinical disease respond to MAP-infected macrophages

**Figure 1:**
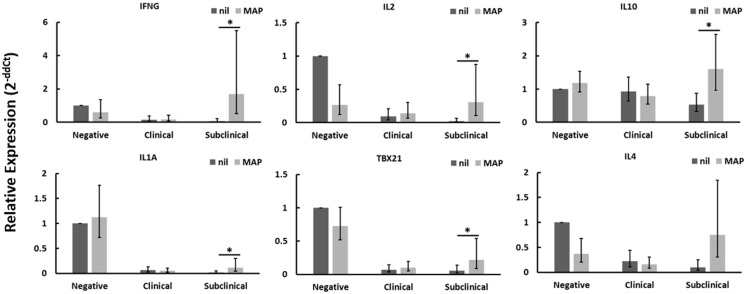
**Relative mRNA abundance of select genes in CD4^+^CD25^−^ T cells cultured with nil- or MAP-infected MDMs**. CD4^+^CD25^−^ T cells were cultured with 4-day old MDMs that were either nil- or MAP-infected. Non-adherent cells were collected following 18-hour incubation. Subsequent RNA extraction and qPCR results are shown. Subclinical infected animals showed up-regulation of a variety of immune genes including those encoding both Th1 (IFNγ, IL1A, TBX21) and Th2 (IL10, IL4) cytokines, whereas clinical infected animals showed no significant changes. *n* = 3–8/group, *p < 0.05.

CD4^+^CD25^−^ T cells from Johne’s test-negative control cows showed a significant reduction in *TGF*β*1* mRNA expression when cultured with MAP-infected MDMs (data not shown) as compared to nil-infected MDMs, but no other significant changes in expression of genes tested were seen. When cultured in contact with MAP-infected MDMs, CD4^+^CD25^−^ T cells from cows with clinical Johne’s disease did not exhibit significant changes, relative to cells in contact with nil-infected MDMs, in the expression of any genes tested. Of the three groups of cows studied, only CD4^+^CD25^−^ T cells from subclinical Johne’s cows showed substantial responses to MAP-infected MDMs when compared to responses to nil-infected (uninfected) MDMs. There was a significant increase in mRNAs encoding *IFN*γ, *IL2*, *IL10*, *IL1A*, and *TBX21* (Figure 1), with a near-significant increase seen in *IL4* (Figure 1) and *TNFA* (data not shown) mRNAs. Of these seven genes, four (*IFN*γ, *IL1A*, *TBX21*, *TNFA*) are characteristic of a Th1 response and two are characteristic of a Th2 response (*IL4*, *IL10*). These data suggest that CD4^+^CD25^−^ T cells from cows with subclinical Johne’s disease respond to MAP antigen presentation on MDMs with a primarily Th1-biased phenotype.

### CD4^+^CD25^−^ T cells from cows with clinical disease are unresponsive and those from cows with subclinical disease show signs of reduced responsiveness

**Figure 2:**
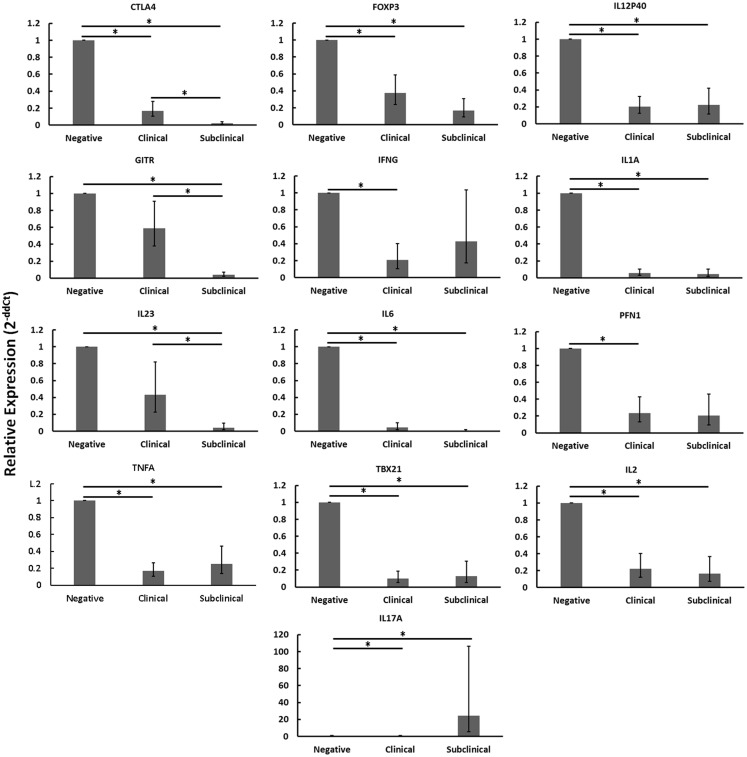
**Relative mRNA abundance of select genes in CD4^+^CD25^−^ T cells cultured with nil- or MAP-infected MDMs, disregarding infection status of MDMs (nil- and MAP-infected MDMs were pooled in this analysis)**. CD4^+^CD25^−^ lymphocytes were cultured with 4-day old MDMs that were either nil- or MAP-infected. Non-adherent cells were collected following 18-hour incubation. Subsequent RNA extraction and qPCR results are shown. Both clinical and subclinical infected animals showed either unchanged expression (data not shown) or downregulation of a variety of immune genes including those encoding Th1, Th2, and Treg proteins. *n* = 3–8/group, *p < 0.05.

When disregarding nil or MAP infection of autologous MDMs, the relative mRNA abundance of numerous immune genes was significantly reduced in CD4^+^CD25^−^ T cells from cows with clinical disease as compared to healthy controls (Figure 2: *CTLA4*, *FOXP3*, *IFN*γ, *IL12P40*, *IL1A*, *IL2*, *IL6*, *PFN1*, *TBX21*, *TNFA*). Similarly, the relative mRNA abundance of several genes was significantly reduced in CD4^+^CD25^−^ T cells of cows with subclinical disease as compared to healthy controls (Figure 2: *CTLA4*, *FOXP3*, *GITR*, *IL12P40*, *IL1A*, *IL2*, *IL23*, *IL6*, *TBX21*, *TNFA*). In both cases, downregulated genes represented a mix of immune phenotypes. While the mRNA encoding the hallmark Th17 cytokine, *IL17*, was significantly up-regulated in CD4^+^CD25^−^ T cells from cows with subclinical disease as compared to both negative control cows and cows with clinical disease, no other genes were significantly up-regulated in either of the groups with Johne’s disease as compared to negative controls. When combined with the generally unchanged expression of most mRNAs studied in CD4^+^CD25^−^ T cells from cows with clinical disease stimulated with MAP-infected MDMs as compared to nil-infected MDMs, it appears probable that CD4^+^CD25^−^ T cells from cows with clinical disease are unresponsive. The fact that mRNA abundance of nearly all genes studied was reduced in CD4^+^CD25^−^ T cells from cows with subclinical disease as compared to healthy controls, combined with the significant increases in various responses to MAP-infected MDMs as compared to nil-infected MDMs in these same animals, suggests that cells from cows with subclinical disease, while still responsive to MAP antigens, are substantially less active than T cells from healthy control cows.

### Expansion of CD4^+^CD25^+^ T cell populations results in an increase in relative Treg abundance in peripheral blood T cell populations from cows with Johne’s disease

**Figure 3:**
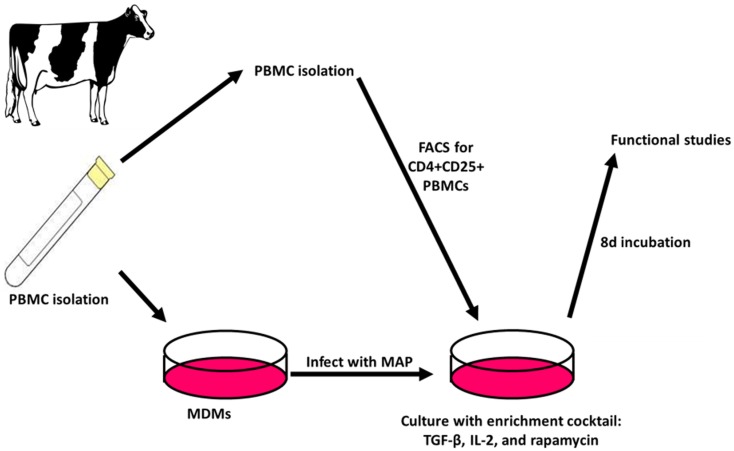
**Treg expansion**. PBMCs are isolated and used to generate MDMs by allowing adherence of monocytes to culture dishes followed by washing away of non-adherent cells. The adherent monocytes were allowed to mature into MDMs for 4 days followed by 24 h infection with MAP. After 3 days, autologous PBMCs are again isolated and FACS is used to select CD4^+^CD25^+^ lymphocytes. The cells are then combined with MAP-infected MDMs and cultured for 8 days with an enrichment cocktail, after which time the expanded CD4^+^CD25^+^ lymphocytes are ready for use in functional studies.

**Figure 4:**
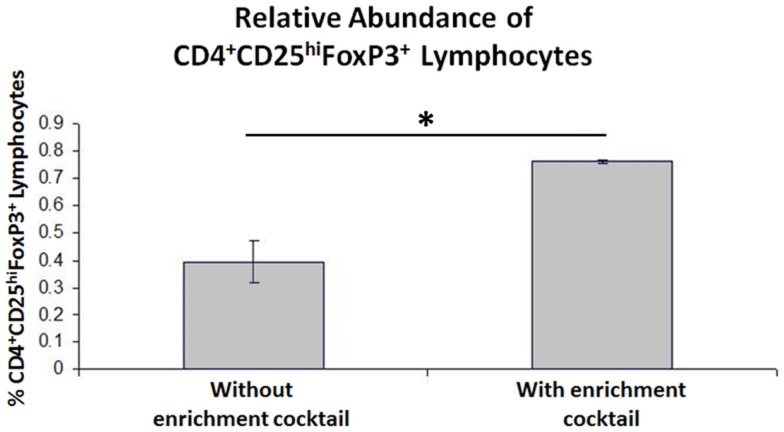
**Results of CD4^+^CD25^+^ lymphocyte expansion**. Three-color flow cytometry was used to measure relative Treg abundance. A successive gating strategy was used to select CD4^+^CD25^hi^FOXP3^+^ lymphocytes. *n* = 4, *p < 0.05.

When the Treg-containing fraction of PBMCs (CD4^+^CD25^+^ lymphocytes) from cows with Johne’s disease (serum ELISA-positive) was cultured with MAP-infected MDMs in the presence of the Treg stimulation cocktail (RPMI complete media supplemented with IL2, TGFβ1, and rapamycin; Figure 3), the relative abundance of Tregs increased significantly, as compared to the same culture conditions without the stimulation cocktail present (Figure 4). Tregs were defined as CD4^+^CD25^hi^FOXP3^+^ lymphocytes.

### MAP stimulation results in a Th1 response by PBMCs

**Figure 5:**
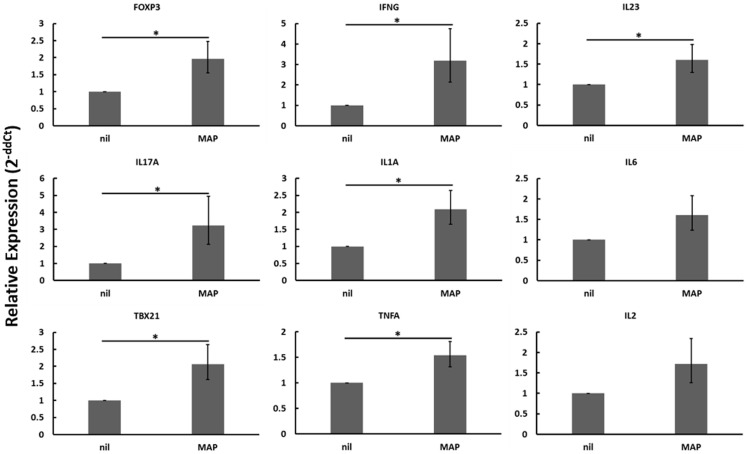
**Relative mRNA abundance of select genes considering the effect of adding MAP to heterogeneous PBMC cell cultures**. When disregarding Johne’s test status or the addition of expanded Tregs (these differing culture conditions were pooled in this analysis), the addition of live MAP resulted in an increase in the relative mRNA abundance of several genes including the Treg-associated FOXP3. mRNAs encoding numerous Th1- (IFNγ, IL1A, TBX21, TNFA) and Th17-associated (IL23, IL17A, IL6) proteins were significantly or near-significantly up-regulated. *n* = 3–8/group, *p < 0.05.

Following Treg expansion (Figure 3), autologous PBMCs from negative control animals and animals in different stages of Johne’s disease were cultured with or without expanded Tregs and with or without live MAP. While we hoped to see Treg-mediated suppression of PBMC responses to MAP as compared to nil stimulation within either the subclinical or clinical infected cow populations, no significant three-way interactions were observed (data not shown). When disregarding Johne’s test status and presence of absence of Tregs, live MAP stimulation resulted in up-regulation of mRNA encoding many genes in the PBMC populations (Figure 5). Overall, up-regulation of both a Th1-like response (*IFN*γ, *IL1A*, *TBX21*, *TNFA*) and a Th17-like response (*IL17A*, *IL23*, *IL6*) was observed; the Th1-like response would be anticipated in negative control cows and cows with subclinical Johne’s disease. It is possible that an unresponsive state of PBMCs from cows with clinical disease resulted in an overall lack of response that may have otherwise dampened the observed Th1- and Th17-like responses upon pooling the test groups as done in the analysis represented in Figure 5.

### Expanded Tregs may not be MAP-specific

**Figure 6:**
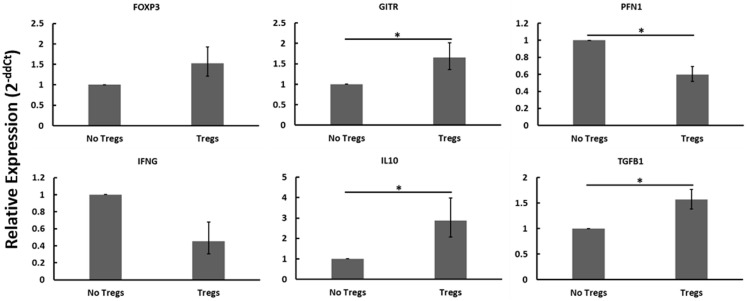
**Relative mRNA abundance of select genes considering the effect of adding expanded Tregs to heterogeneous PBMC cell cultures**. When disregarding Johne’s test status and nil versus MAP stimulation (these conditions were pooled in this analysis), the addition of expanded Tregs resulted in a significant increase in mRNA expression of several Treg-associated genes including FOXP3, GITR, IL10, and TGFβ1. It also resulted in a significant decrease in two Th1 cytokines, IFNγ and PFN1. *n* = 3–8/group, *p < 0.05.

Autologous PBMCs were cultured with or without expanded Tregs and with or without live MAP. When disregarding Johne’s test status and the presence or absence of live MAP, the addition of Tregs resulted in notable changes to the gene expression profile that are consistent with Treg function and activity (Figure 6). Namely, the relative abundance of mRNAs encoding Treg-associated genes (*FOXP3*, *GITR*, *IL10*, *TGF*β*1*) all increased, with only *FOXP3* not reaching significance (*p* = 0.0764). Further, and critical to the argument in favor of the functionality of the expanded Tregs, the relative mRNA abundance of Th1-associated factors *PFN1* (which encodes perforin, a protein key to cytotoxic T cell cytolytic function) and *IFN*γ decreased when Tregs were added to the cultures. Due to the apparent presence and activity of the expanded Tregs, while considering the lack of significant interaction when specifically examining different test statuses or nil versus MAP stimulation, it seems probable that the expanded Tregs may not all be MAP-specific as initially anticipated.

## Discussion

Regulatory T cells have been suggested as a potential player in the progression of Johne’s disease in previous studies (10, 16, 24). Evidence has shown that Tregs likely do not play a role in establishing infection (25) but rather, may facilitate the shift between Th1 and Th2 immune responses (10, 16) indicative of the transition from subclinical to clinical disease. Two conditions proposed to promote the development of Tregs are ineffective co-stimulation (18–20) and chronic antigen stimulation (5, 16). Both of these conditions are encountered during the course of Johne’s disease (5, 17). Further, it has been shown that removal of either CD4^+^ or CD25^+^ cell populations from PBMCs of cows with Johne’s disease prior to stimulation with MAP antigens results in a decrease in *IL10* mRNA abundance and an increase in *IFN*γ mRNA abundance (10), suggesting that a population of Tregs may be present and responsive to MAP antigens.

Based on the above evidence, in the present study, we sought to determine if peripheral CD4^+^CD25^−^ T cells from cows in different stages of Johne’s disease would express a Treg phenotype upon exposure to MAP-infected macrophages (MDMs). While we anticipated the induction of a Treg phenotype in CD4^+^CD25^−^ T cells from cows with clinical disease upon exposure to MAP-infected MDMs, we observed an unexpected outcome. In fact, relative mRNA abundance of all genes observed (exception: *IL17A*) either decreased or remained unchanged in all cows with Johne’s disease (regardless of disease stage) when compared to healthy controls. In many ways, these CD4^+^CD25^−^ T cells appeared to show signs of unresponsiveness. One possible reason for this observed unresponsiveness is T cell anergy, although the experiments described here are not sufficient for making such a connection. T cell anergy is a state of functional inactivation following antigen stimulation under non-optimal conditions (26); one of these conditions is often the absence of co-stimulation (27, 28). Additionally, T cells are generally non-responsive to antigen stimulation (26) and it is in response to MAP antigens presented on the macrophage surface that we see key differences between cows with subclinical and clinical disease, providing one possible indicator of anergy (although further experiments are needed to truly claim anergy as the reason for the observed unresponsiveness).

Our data show that CD4^+^CD25^−^ T cells from cows with subclinical disease, while showing generally reduced gene expression compared to similar cells from negative control cows, do still respond to stimulation with MAP-infected MDMs with a primarily Th1 profile (Figure 1) as well as up-regulating mRNA encoding IL2, when compared to stimulation with nil-infected MDMs. These data support the notion that peripheral CD4^+^CD25^−^ T cells from cows with subclinical disease do respond to MAP antigen encounter in an appropriate Th1 manner; however, the generally decreased profile of immune gene expression suggests that these cells are simply less active overall when compared to T cells from healthy control cows. In contrast, CD4^+^CD25^−^ T cells from cows with clinical disease did not significantly up-regulate mRNA of any genes studied when presented with MAP antigens via MDMs, as compared to presentation with nil-infected MDMs. This combined with the generally reduced mRNA profile of these cells when compared to those of healthy control cows suggests that peripheral CD4^+^CD25^−^ T cells from cows with clinical disease are almost completely unresponsive. Unfortunately, the cows used in this study were observed only once. Future studies should focus on repeating these experiments while following one set of cows through progression of Johne’s disease, importantly during the transitions from uninfected to subclinical disease, and from subclinical to clinical disease. Indeed, this would allow determination of whether or not CD4^+^CD25^−^ T cells in cows infected with MAP initially respond to the pathogen appropriately, but over time develop an unresponsive phenotype, allowing progression into clinical disease. Such experiments should also be conducted with experimentally infected cows.

In our second set of experiments, we initially developed a means to increase the relative abundance of Tregs in a CD4^+^CD25^+^ T cell population. Our Treg expansion assay was developed with the intention of expanding MAP antigen-reactive Tregs specifically. As mentioned previously, MAP colonization of macrophages results in reduced co-stimulation (17). Therefore, our assay created conditions that favored induction of a Treg phenotype. In addition, we added three exogenous factors (IL2, TGFβ1, rapamycin) that have been shown to expand the Treg population of human cord blood (29). As shown in Figure 4, our assay successfully expanded the relative abundance of Tregs in CD4^+^CD25^+^ T cell populations from cows with Johne’s disease. We anticipated that these Tregs might be MAP antigen-reactive, and therefore, sought to use them in functional assays. For the functional assays we took PBMCs from three different groups of cows (healthy controls, cows with subclinical disease, and cows with clinical disease) and combined them with or without live MAP and with or without autologous expanded Tregs.

Prior to beginning our experiments, we anticipated finding Treg-mediated suppression of Th1 immune responses to MAP stimulation in PBMCs from cows with clinical disease. We found that this was not the case, and these results are in line with what was observed when peripheral CD4^+^CD25^−^ T cells from cows with clinical disease were brought in contact with MAP-infected MDMs. If, as demonstrated in the first set of experiments, CD4^+^CD25^−^ T cells from cows with clinical disease are unresponsive, then it stands to reason that Tregs taken from these same animals would not suppress immune responses to MAP, and in fact this is what was observed in our second set of experiments. As we demonstrated small but significant responses to MAP-infected MDMs in peripheral CD4^+^CD25^−^ T cells from cows with subclinical disease, we expected to find Treg-mediated suppression of PBMC responses to MAP in cells from cows with subclinical disease. Unexpectedly, we did not observe Treg-mediated suppression of PBMC responses to MAP in subclinical cows as we had anticipated. This may have been due in part to small sample size. Overall, no significant three-way interactions (interactions comparing Johne’s disease test status, presence or absence of MAP, and presence or absence of expanded Tregs) were observed for any of the genes analyzed. One possible reason for this may be that our study groups were too small to reach significance with certain genes when working with an outbred species, such as cattle. As such, we tested for significance of any one- or two-way interactions within our data sets in an effort to glean further information from our experiments. The fact that we reached significance with many genes when looking at one-way interactions, upon disregarding the other factors, suggests that greater sample sizes may yield further insights into Treg activity in cows with Johne’s disease.

Despite these unexpected results, upon examining overall one-way interactions significance was observed in many cases. For instance, when only considering nil or MAP stimulation (and thus, grouping all samples based on nil or MAP stimulation alone), there was strong evidence suggesting a mixed Th1/Th17 response to live MAP in bovine PBMCs as expression of the Th1-associated genes *IFN*γ, *IL1A*, *TBX21*, and *TNFA* and the Th17-associated genes *IL23*, *IL17A*, and *IL6* were all significantly (exception: IL6, *p* = 0.076) upregulated. This demonstrates an attempt by PBMCs from all animals, regardless of Johne’s disease test status, to respond to MAP antigens. The Th17 response, while not unprecedented in Johne’s disease (30, 31), may be indicative of an immune response gone astray. Critically, expression of *FOXP3* mRNA was also significantly upregulated in the presence of MAP as compared to nil-stimulated samples, suggesting a role for MAP in inducing expression of *FOXP3* mRNA in bovine PBMCs.

When we compared the presence of Tregs to the absence of Tregs regardless of Johne’s test status or MAP stimulation, we found that numerous Treg-associated factors (*FOXP3*, *GITR*, *IL10*, *TGF*β*1*) were indeed up-regulated when expanded Tregs were added to autologous PBMC cultures. While this confirms our flow cytometric findings that Tregs were being expanded in our expansion assay, it also suggests that these Tregs may not be MAP antigen-reactive as we had anticipated. A possible discrepancy arises when considering that mRNA encoding IL10 and TGFβ1 did not up-regulate in response to MAP antigens but did up-regulate when expanded Tregs were added to cultures generally, whereas *FOXP3* mRNA was upregulated under both conditions. It may be that MAP is important in inducing expression of FOXP3 and generation of Tregs, but that these Tregs do not respond specifically to MAP via production of IL10 or TGFβ1. Further, if MAP was key to expanding MAP-antigen-specific Tregs, then there would not have been an expansion of Tregs in cultures from test-negative cows and thus any significant increases in abundance of Treg-associated genes would have been seen only in cows with Johne’s disease. In reality, however, we found that significant increases in Treg-associated genes were only seen when we grouped results from all different Johne’s disease test statuses together. Overall, it appears that the expansion protocol did not appear to expand MAP-reactive Tregs uniquely; rather it is possible that the stimulation cocktail alone was sufficient to induce expansion of non-MAP-reactive Tregs as well. It has been shown that memory Tregs proliferate less well (32), and therefore, in this context it is unsurprising that a MAP-specific suppressive effect was not detected. Future Treg studies will focus on MAP-containing lesions in cows with Johne’s disease in an effort to identify MAP-specific effects.

Finally, although the expanded Tregs may not be antigen-specific, our results do suggest that they are functional, and that they are functioning regardless of the presence or absence of MAP. When considering only the presence or absence of Tregs, we found that there is a decrease in the expression of two key Th1 genes, *IFN*γ and *PFN1*, in the presence of expanded Tregs. As Tregs are known to exert immunosuppressive effects on Th1 cytokine expression (33, 34), we believe that these data demonstrate that expanded Tregs are both non-antigen-specific and are functional. Although the Tregs appeared to be constitutively functional *in vitro* (exerting a suppressive effect on Th1 cytokine expression regardless of antigenic stimulation), whether or not they require antigenic stimulation *in vivo* remains unclear. Future studies should focus on increasing cow numbers to better reach significance when considering such complex interactions. Regulatory T cell expansion, although promising as shown here, should be further investigated to determine the exact functional nature of the expanded Tregs, perhaps with consideration toward using them in an *in vivo* model of MAP infection and Johne’s disease. Finally, it will be of critical importance to examine the existence or presence of Tregs within lesions caused by MAP during various stages of Johne’s disease. If Tregs are found, it will be critical to determine what if any function they may serve within these lesions.

## Conflict of Interest Statement

The authors declare that the research was conducted in the absence of any commercial or financial relationships that could be construed as a potential conflict of interest.
